# Supplementary material for: Optogenetic rewiring of thalamocortical circuits to restore function in the stroke injured brain
Source: Nat Commun. 2017 Jun 23;8:15879. doi: 10.1038/ncomms15879 (PMC5490053; doi:10.1038/ncomms15879)
Supplement: Supplementary Information [file ncomms15879-s1.pdf]

Type of file: PDF
Size of file: 0 KB
Title of file for HTML: Supplementary Information
Description: Supplementary Figures

Type of file: PDF
Size of file: 0 KB
Title of file for HTML: Peer Review File
Description:

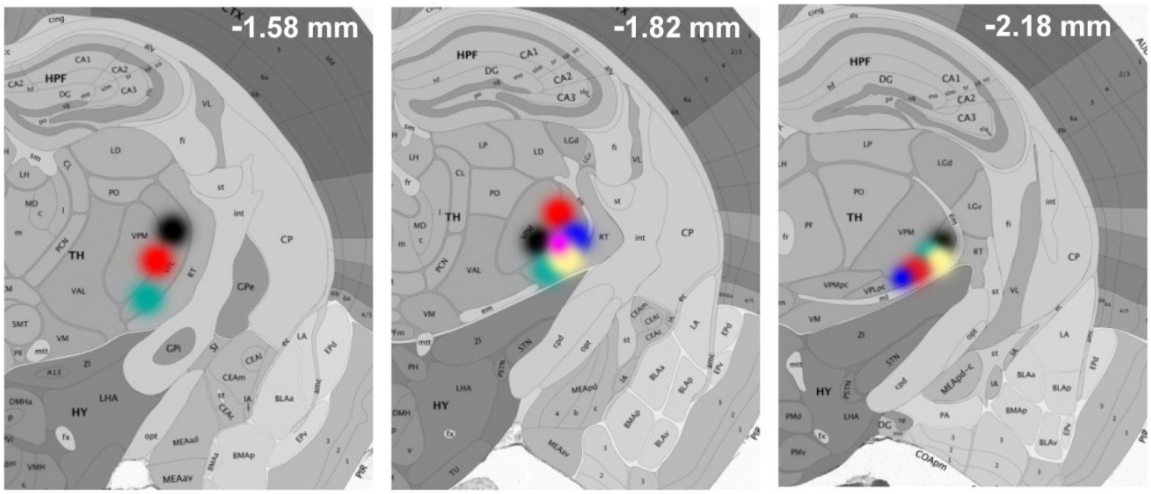

Supplementary Figure 1. Summary of AAV injection sites. Thalamic injection sites from 14 different mice are shown with colors in the 3 coronal sections (images from Allen Mouse Brain Atlas, http://mouse.brain-map.org/experiment/thumbnails/100048576?image_type=atlas). Approximate coronal section position relative to bregma is shown in top right corner of each image.

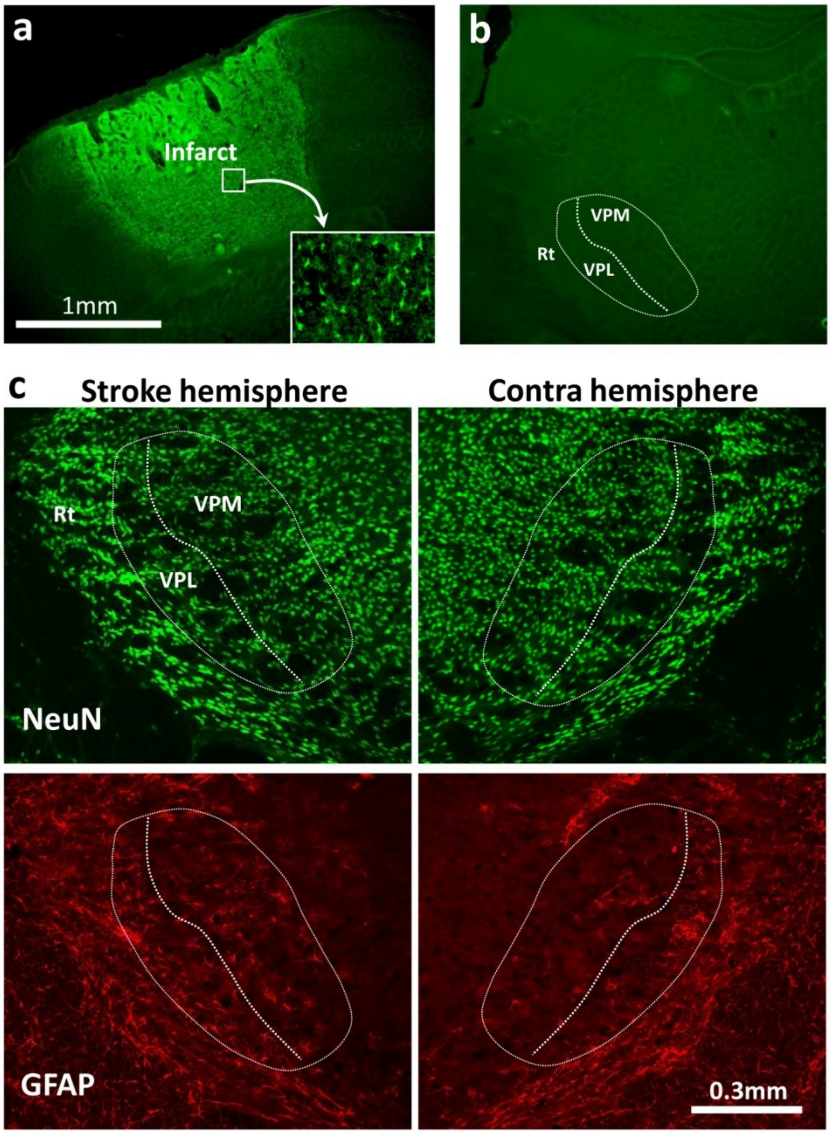

Supplementary Figure 2. No obvious signs of neuronal degeneration and death in the thalamus after focal stroke Images show Fluoro-jade C staining in the cerebral infarct four days after stroke (a), and in the ipsilateral thalamus (b). (c) Confocal images showing NeuN or GFAP staining in the thalamus ipsilateral or contralateral to the cortical stroke. Rt: reticular thalamic nucleus; VPM: ventral posterior medial nucleus; VPL: ventral posterior lateral nucleus.

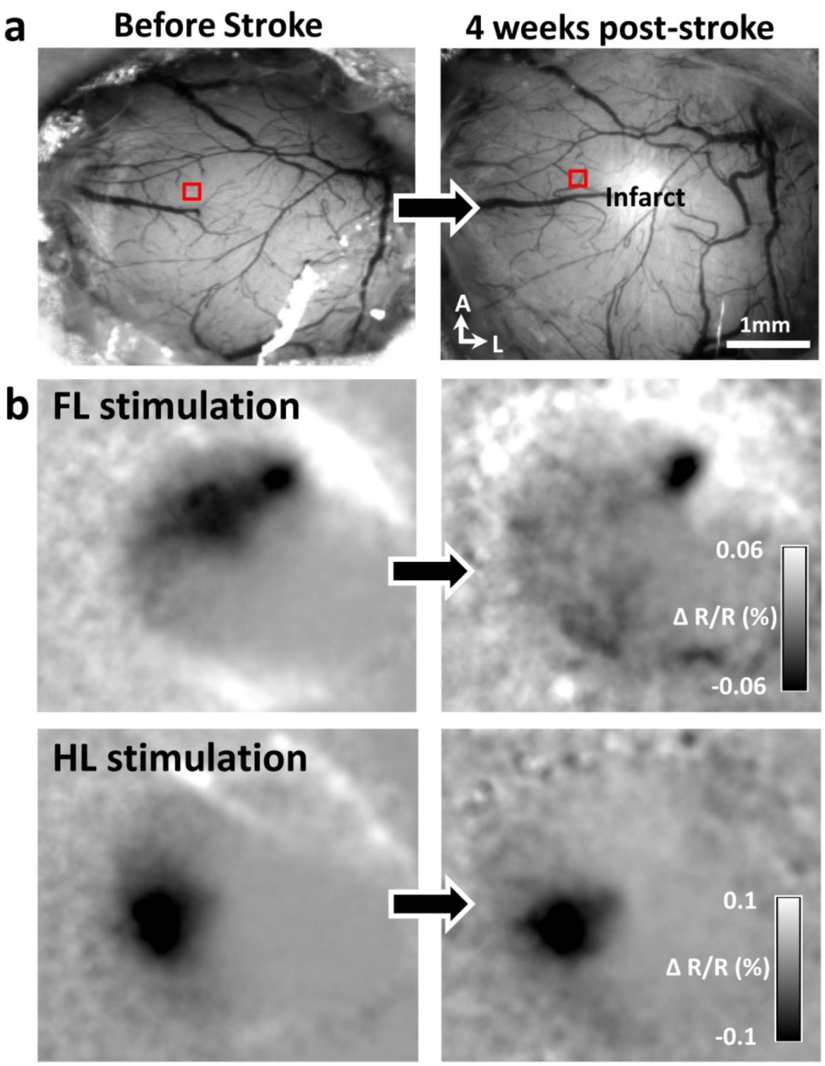

Supplementary Figure 3. Forelimb and hindlimb cortical representations before and after stroke. (a) Brightfield images of cortical surface before stroke and 4 weeks afterwards. In this particular example, calcium responses from thalamocortical boutons were imaged in the peri-infarct region denoted by the red boxes. (b) Images show forelimb and hindlimb evoked cortical responses before stroke (left column) and 4 weeks later (right column).

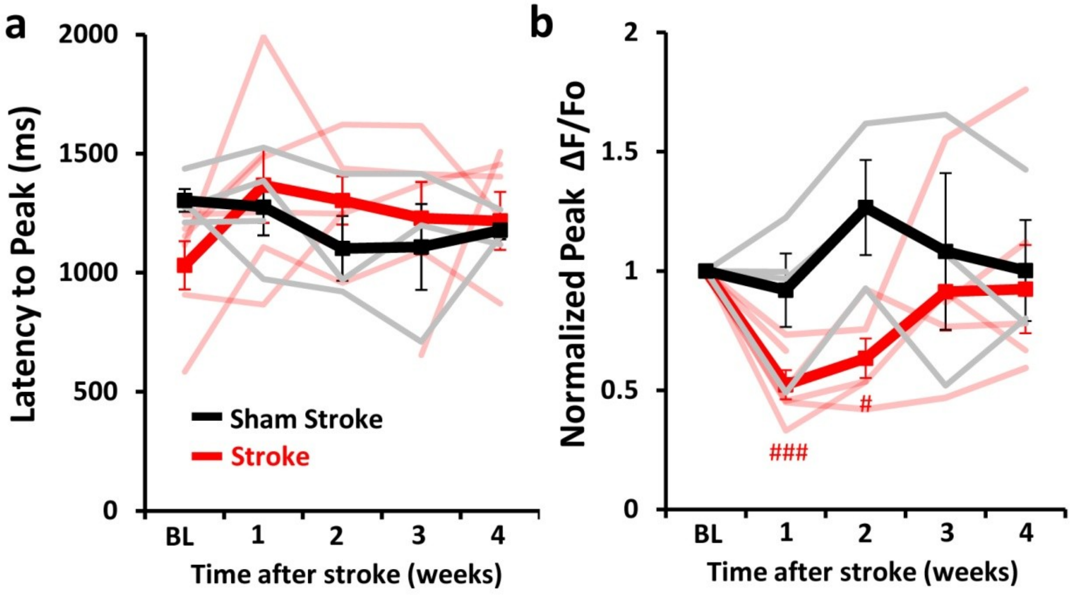

Supplementary Figure 4. Effects of stroke or sham surgery on thalamocortical bouton responses. Graphs show the latency (a) and normalized peak amplitude (b) of calcium responses in thalamocortical boutons before stroke or sham procedure and up to 4 weeks afterwards. Group averages are shown with thick lines, while individual animal plots are represented by faint, thin lines. #$p<0.05$, ###$p<0.001$ based on one sample t-tests to baseline. Data are means ± S.E.M.

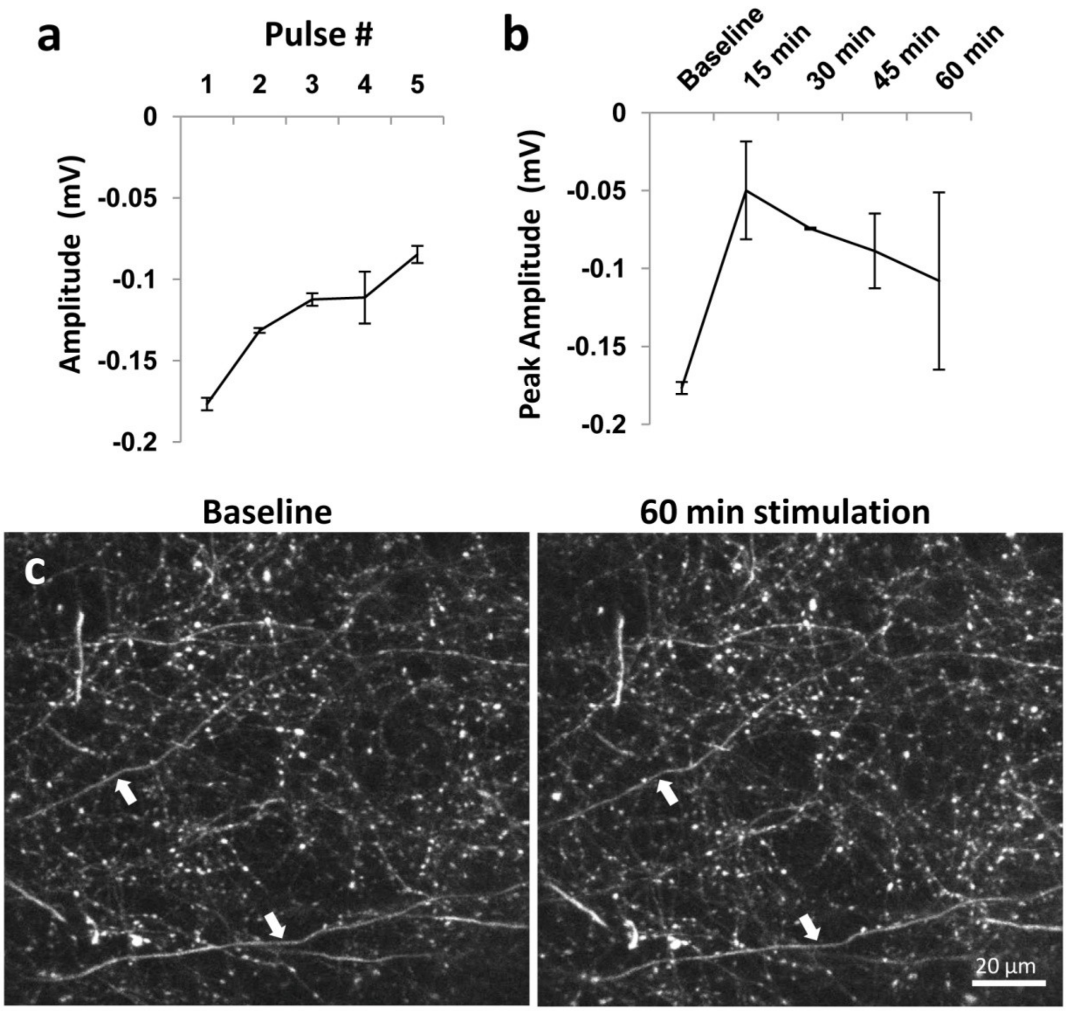

Supplementary Figure 5. Effects of stimulation on cortical field potentials and thalamocortical axon structure. (a) Graph shows the peak amplitude of blue light evoked cortical field potentials with each successive pulse in first 5 Hz train. (b) Graph shows average peak cortical responses to 5 Hz blue light stimulation at 15 minute intervals over the 60 minute stimulation session. (c) Maximal intensity z-projection images of thalamocortical axons before (left) and after (right) 60 minutes of intermittent 5Hz optical stimulation. Data are means ± S.E.M.

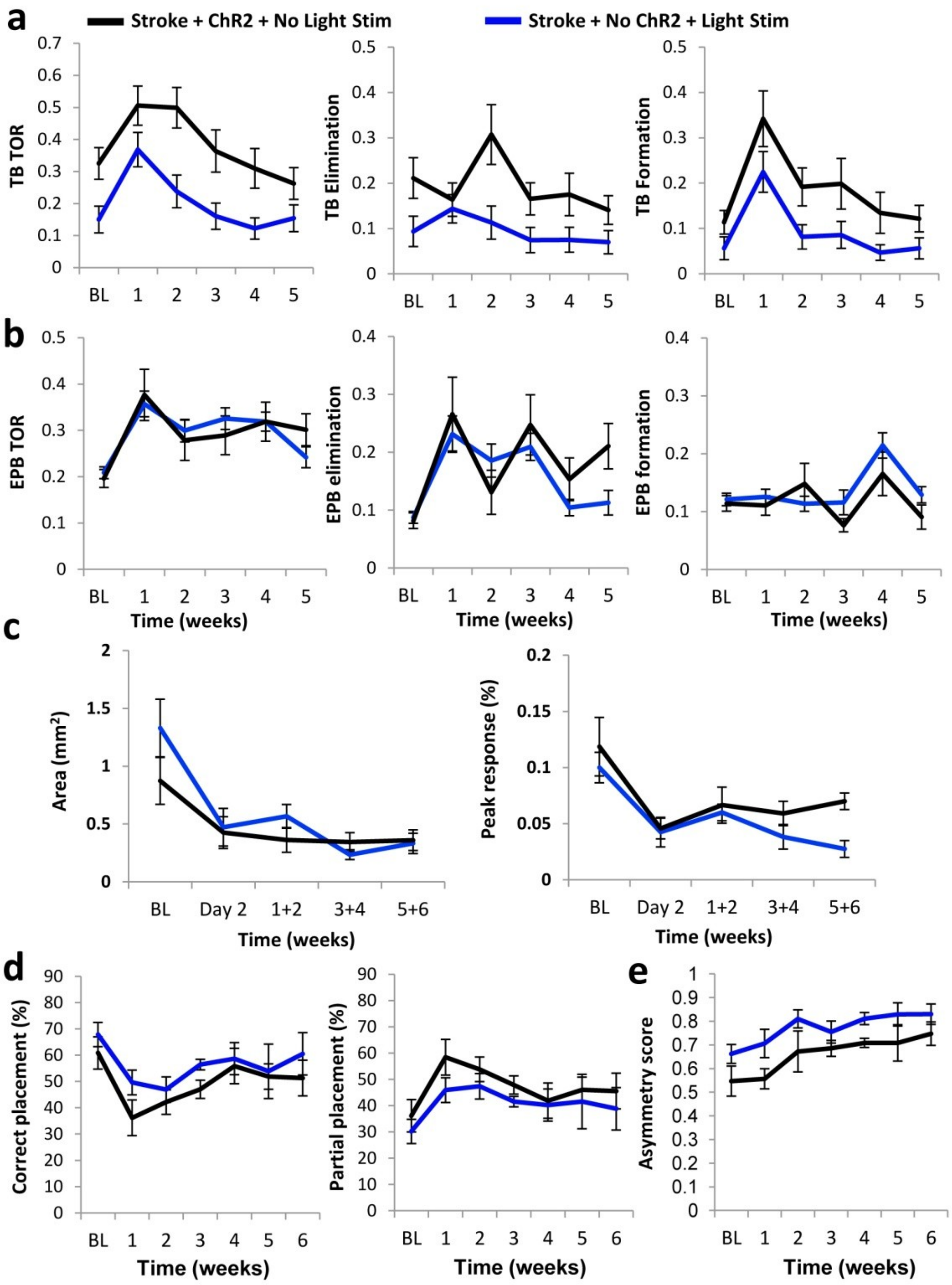

Supplementary Figure 6. Comparison of stroke control stimulation groups on axonal bouton dynamics, sensory evoked cortical responses and forepaw function. Graphs plot TB (a) and EPB dynamics (b), the area and amplitude of forelimb evoked cortical responses (c), and behavioural results in the horizontal ladder (d) and tape removal test (e) in stroke affected mice that comprise the control stimulation group. Control stimulation mice received either blue light without ChR2 expression (purple lines, n=4 mice) or ChR2 expression with no blue light (black lines, n=7 mice). Data are means ± S.E.M.

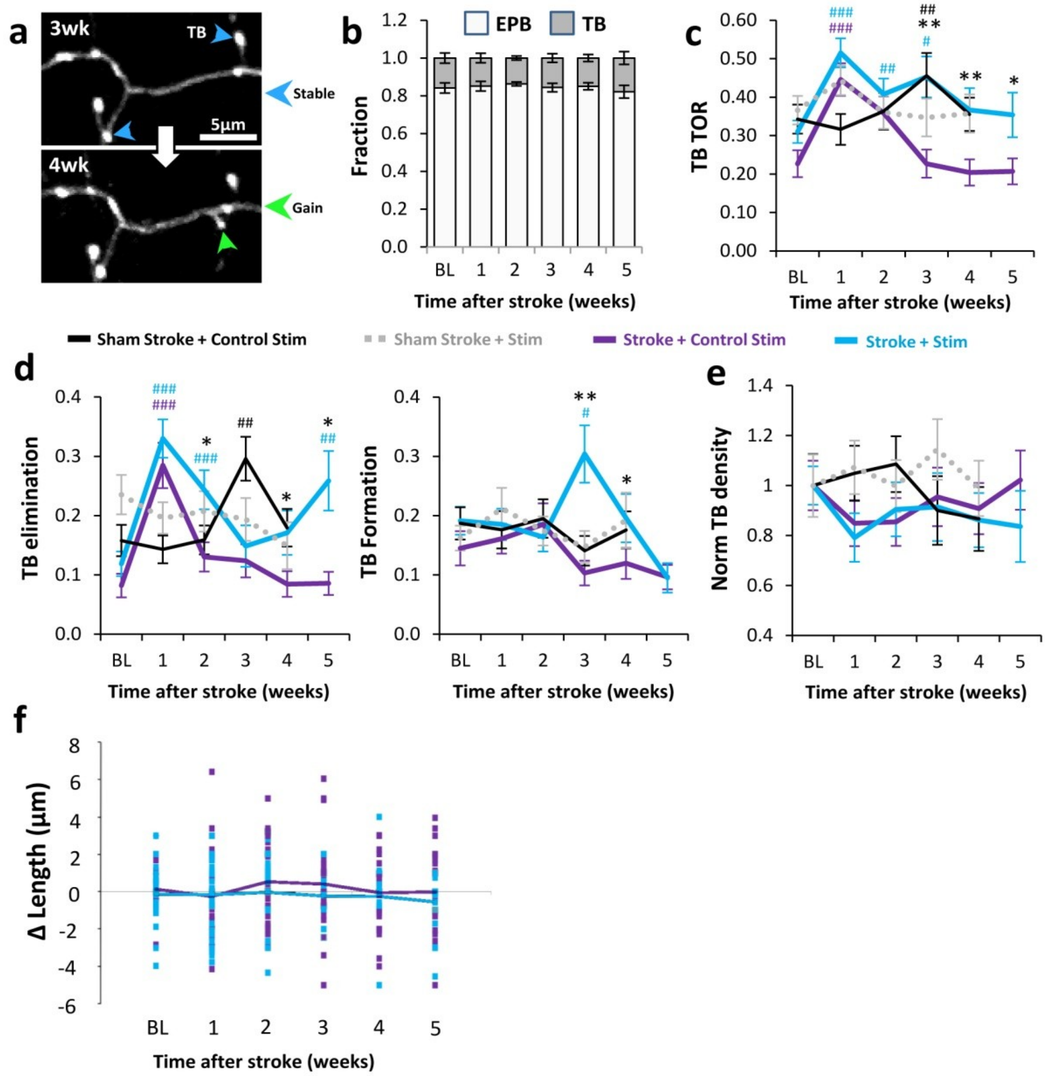

Supplementary Figure 7. Terminaux bouton dynamics after stroke (a) Maximal z projection images showing TB dynamics from 3 to 4 weeks recovery. (b) Fraction of axonal boutons classified as either TB or EPB. Both stroke groups were combined in this figure. (c) Graph plotting weekly TB turnover ratio (TOR) in: i) sham stroke receiving control stimulation (solid black line, n=5 mice, 125 axons, 288 TB); ii) sham stroke with optogenetic stimulation (dashed grey line, n=6 mice, 103 axons, 251 TB), iii) stroke with optogenetic stimulation (teal line; n=6 mice, 84 axons, 253 TBs) and iv) stroke with control stimulation (purple line; n=6 mice, 97 axons, 193 TBs). (d) Fraction of TBs eliminated or formed from week to week. (e) Graph plotting the density of thalamocortical TBs normalized to baseline values. (f) Graph plotting changes in length of TBs from week to week in stroke affected mice with or without optogenetic stimulation. The average change in TB length is shown with solid lines for each group of mice. Data are means ± S.E.M. #$p<0.05$, ##$p<0.01$, ###$p<0.001$ based on t-tests comparing post-stroke time points in each group designated by color to respective baseline values. *$p<0.05$, **$p<0.01$, ***$p<0.001$ based on t-tests comparing stroke+stimulation vs. stroke+control stimulation.

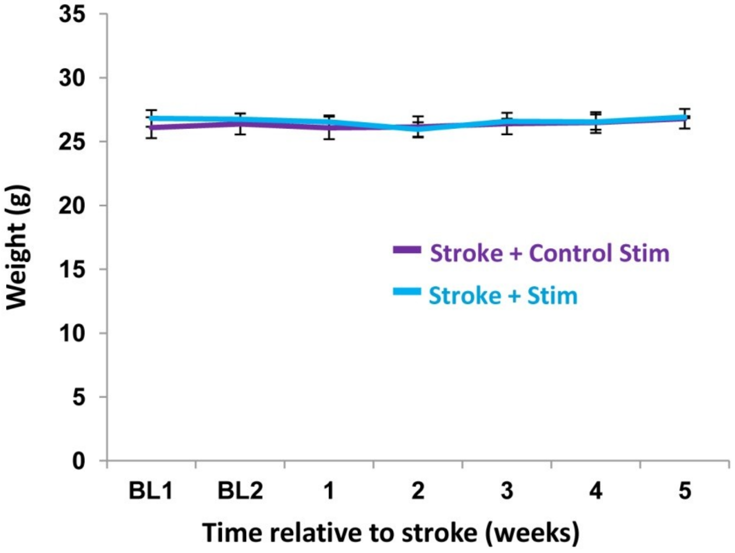

Supplementary Figure 8. Graph shows body weights from mice receiving stroke and optogenetic or control stimulation. Data are means ± S.E.M.
